# A Perikinetochoric Ring Defined by MCAK and Aurora-B as a Novel Centromere Domain

**DOI:** 10.1371/journal.pgen.0020084

**Published:** 2006-06-02

**Authors:** María Teresa Parra, Rocío Gómez, Alberto Viera, Jesús Page, Adela Calvente, Linda Wordeman, Julio S Rufas, José A Suja

**Affiliations:** 1 Departamento de Biología, Universidad Autónoma de Madrid, Madrid, Spain; 2 Department of Physiology and Biophysics, School of Medicine, University of Washington; Seattle, Washington, United States of America; Stowers Institute for Medical Research, United States of America

## Abstract

Mitotic Centromere-Associated Kinesin (MCAK) is a member of the kinesin-13 subfamily of kinesin-related proteins. In mitosis, this microtubule-depolymerising kinesin seems to be implicated in chromosome segregation and in the correction of improper kinetochore-microtubule interactions, and its activity is regulated by the Aurora-B kinase. However, there are no published data on its behaviour and function during mammalian meiosis. We have analysed by immunofluorescence in squashed mouse spermatocytes, the distribution and possible function of MCAK, together with Aurora-B, during both meiotic divisions. Our results demonstrate that MCAK and Aurora-B colocalise at the inner domain of metaphase I centromeres. Thus, MCAK shows a “cone”-like three-dimensional distribution beneath and surrounding the closely associated sister kinetochores. During the second meiotic division, MCAK and Aurora-B also colocalise at the inner centromere domain as a band that joins sister kinetochores, but only during prometaphase II in unattached chromosomes. During chromosome congression to the metaphase II plate, MCAK relocalises and appears as a ring below each sister kinetochore. Aurora-B also relocalises to appear as a ring surrounding and beneath kinetochores but during late metaphase II. Our results demonstrate that the redistribution of MCAK at prometaphase II/metaphase II centromeres depends on tension across the centromere and/or on the interaction of microtubules with kinetochores. We propose that the perikinetochoric rings of MCAK and Aurora-B define a novel transient centromere domain at least in mouse chromosomes during meiosis. We discuss the possible functions of MCAK at the inner centromere domain and at the perikinetochoric ring during both meiotic divisions.

## Synopsis

The centromere is a chromosome domain essential for the correct partitioning of chromosomes during mitotic and meiotic cell divisions. MCAK is a centromeric protein that depolymerises microtubules, and seems to be implicated in chromosome segregation, and in the correction of improper microtubule interactions with the chromosome. However, there are no published data on its behaviour and function during meiotic divisions. Here, Parra et al. analyse the pattern of distribution of MCAK during male mouse meiosis in relation to Aurora-B, a kinase that regulates its activity. They show that MCAK and Aurora-B appear at the inner domain of metaphase I bivalents and unaligned metaphase II chromosomes. Most importantly, the authors discovered that these proteins relocalise to a novel perikinetochoric ring in aligned metaphase II chromosomes. The discovery of this novel structure adds a new dimension to the understanding of kinetochore structure and biology. The authors propose that, at least for mouse centromeres, the perikinetochoric ring represents a transient centromere domain whose appearance depends on tension across centromeres once microtubules interact with both sister kinetochores. This study shows that the analysis of the behaviour of different centromere proteins during meiosis can offer new insights concerning the centromere functionality.

## Introduction

The centromere is a structural domain of mitotic and meiotic chromosomes essential for their correct segregation at cell division. This domain is composed of the kinetochore and the subjacent chromatin. The kinetochore is located on the outside face of the centromere, and is composed of several distinct layers. There is an inner plate, constituted by chromatin containing nucleosomes with at least centromeric protein A (CENP-A), a specialised histone H3 variant, auxiliary proteins, and DNA [1]. Additionally, there is an outer plate mainly composed of microtubule (MT) motor proteins which are involved in chromosome alignment during prometaphase and chromatid segregation at anaphase, and mitotic spindle checkpoint proteins that are involved in the regulation of the metaphase/anaphase transition [2].

The region between the two sisters kinetochores is called the inner centromeric domain which has been defined as the interaction place between sister chromatids at metaphase chromosomes [3]. This domain was firstly defined by the location of chromatid linking proteins (CLiPs) [4] and inner centromeric protein (INCENP), a chromosomal passenger protein [5]. The cohesin subunit RAD21 has also been localised at this domain [6]. Besides INCENP, the others components of the chromosomal passenger proteins complex: Aurora-B, survivin, and recently Borealin/Dasra and Aurora-C, are also localised at the inner domain [7,8]. This complex has been implied in several cell division processes such as chromatin modification, spindle assembly, the completion of cytokinesis and the correction of kinetochore attachment errors [8]. The first suggestion of the involvement of the passenger complex in kinetochore attachment errors came from the experiments in yeast where mutants for homologues of Aurora-B and INCENP were unable to biorientate their chromosomes [9]. In HeLa cells the inhibition of Aurora-B by hesperadin caused an elevated frequency of chromosomes showing an abnormal syntelic orientation [10].

The MT-depolymerising kinesin mitotic centromere-associated kinesin (MCAK) is also localised to the inner centromere domain. This protein is detected at mammalian mitotic centromeres from late prophase up to late telophase [11], and is thought to be implicated in chromosome segregation. This protein is a member of the kinesin-13 subfamily of kinesin-related proteins that shares a high homology with other member of the kinesin superfamily [11–13]. However, the kinesin-13 family uses the hydrolysis of ATP to depolymerise MTs from either end [14–16]. This activity plays a critical role in the assembly and function of the mitotic spindle. Thus, in *Xenopus* egg extracts, depletion of XKMC1, the *Xenopus* orthologue of MCAK, decreases the catastrophe rate of MT ends and causes chromosomes to misalign on the mitotic spindle [17,18]. In mammalian cells, depletion of MCAK by antisense DNA interferes with anaphase chromosome segregation [19]. Additionally, recent studies have demonstrated that MCAK disruption by injecting a dominant negative protein into prophase, led to reduced centromere stretch, delayed chromosome congression, alignment defects, and severe missegregation of chromosomes [20]. Moreover, the disruption of MCAK leads to multiple kinetochore-MT attachments. These data suggest that MCAK is required for correction of improper kinetochore-MT interactions [20].

Since Aurora-B and MCAK share a common distribution at the inner centromeric domain, it was reasonable to think that they could be interacting. In fact, Aurora-B seems to regulate the activity of MCAK [21–23]. In this sense, Aurora-B appears to have two different functions. First, Aurora-B is required for MCAK loading at centromeres. Second, Aurora-B phosphorylates MCAK and inhibits its MT-depolymerisation activity. A comparison of the distribution of Aurora-B and MCAK in mono-oriented chromosomes lacking bipolar attachment showed that sister kinetochores were close together, and that the distribution of MCAK and Aurora-B overlapped completely. When chromosomes were bioriented at the metaphase plate Aurora-B remained in the inner centromere but MCAK redistributed beneath kinetochores. These results suggest that the distribution and activity of MCAK may be regulated by tension across centromeres exerted by MTs [21]. Moreover, it has been hypothesised that tip tracking along kinetochore MTs (kMTs) by dephosphorylated MCAK may be the mechanism by which active MCAK leaves the inner domain and loads at the outer centromere [24]. This hypothesis led to think that a subcellular regulation of MCAK would be an ideal mechanism to release inappropriate MTs binding [25].

Recently, another protein has been implicated in preventing kinetochore-MT attachment errors. Inner centromere kin/ stimulator (ICIS) is a protein that targets to centromeres in a MCAK-dependent manner, and that stimulates MCAK in vitro [26]. It has been proposed that MCAK-ICIS may destabilise MTs associated laterally with inner centromeres [26]. Moreover, these two proteins coimmunoprecipitate with INCENP and Aurora-B in *Xenopus* extracts [9].

Most studies have analysed the role of MCAK in mitosis, but there are no studies describing the distribution and behaviour of MCAK in meiosis, where two divisions occur after a single round of DNA replication. In this study we have analysed for the first time the subcellular distribution of MCAK on mouse spermatocytes during both meiotic divisions. For this purpose, we made a double immunolabelling of MCAK and SYCP3, a protein component of the lateral elements (LEs) of the synaptonemal complex, to precisely analyse the appearance and behaviour of MCAK depending on the different meiotic stages. Double immunolabellings of MCAK with kinetochores and the kinase Aurora-B allowed us to study their relative distributions during chromosome congression to the metaphase I and II plates. Also, to test whether there is any relationship between MCAK distribution and the kinetochore-MT attachment to metaphase II centromeres, we analysed MCAK altogether with α-tubulin and BubR1, a spindle checkpoint protein, and the MCAK distribution in colchicine-treated spermatocytes. Finally, we studied the ultrastructure of metaphase II centromeres by electron microscopy conventional techniques, and by employing the osmium tetroxide/p-phenylenediamine (Os-PPD) cytochemical technique that preferentially detects ribonucleoproteins. We discuss the possible functions of MCAK and Aurora-B during both meiotic divisions.

## Results

### MCAK Is Recruited at Centromeres after Aurora-B during Late Diplotene

**Figure 1:**
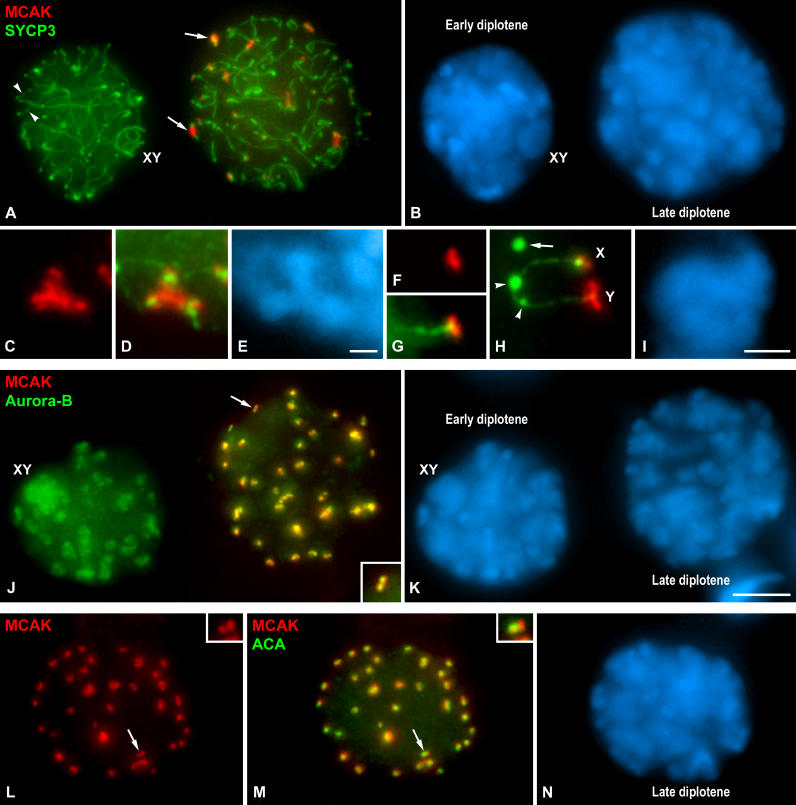
MCAK Is Loaded at Centromeres after Aurora-B in Late Diplotene MCAK is labelled in red and SYCP3 (A–I), Aurora-B (J,K), and kinetochores (L–N) are labelled in green. In merged images colocalisation regions appear in yellow. DNA appears in blue. Most images are z-projections of several focal planes throughout the spermatocytes shown. (A and B) Early and late diplotene spermatocytes. At early diplotene SYCP3 labels desynapsing LEs (arrowheads in [A]), and the X and Y axial elements (XY). At this stage no MCAK labelling can be detected. Nevertheless, during late diplotene, when most LEs are desynapsed, MCAK can be detected as small dots at one of their ends (arrows in [A]). (C–E) Single focal plane of a late diplotene chromocentre. MCAK occupies part of the heterochromatin and colocalises partially with the proximal ends of three desynapsed LEs detected with SYCP3. (F and G) Single focal plane of the centromeric region of a late diplotene autosome. MCAK is detected as two dots at the end of a desynapsed LE. (H and I) Projection of three focal planes of the sex bivalent (X,Y) in diakinesis. While the Y centromere appears scarcely stained for SYCP3, MCAK shows an intense T-like labelling. By contrast, the MCAK labelling at the X centromere is fainter. Note that there is one SYCP3 agglomerate in the nucleoplasm (arrow), and two SYCP3 accumulations on the axial elements (arrowheads). (J and K) Early and late diplotene spermatocytes. At early diplotene, Aurora-B appears at chromocentres and the sex body (XY). MCAK becomes first detectable at centromeres in late diplotene mostly colocalising with Aurora-B. The arrow indicates the enlarged region shown in the inset. (L–N) Late diplotene spermatocyte. The MCAK signals appear close to the kinetochore ones, that have been revealed with an anti-centromere autoantibody serum. The arrowed signal has been enlarged in the inset where it can be discerned that a single kinetochore is located in between two MCAK signals. Bars, 5 μm (A,B,J,K,L–N), 1 μm (C–E), and 2 μm (F–I).

To precisely determine the appearance and behaviour of MCAK during prophase I, we performed a double immunolabelling of MCAK and SYCP3 on squashed spermatocytes (Figure 1). We used a labelling with SYCP3, a structural protein component of LEs of the synaptonemal complex, since it allowed us to discriminate among the different prophase I stages. Our results showed that MCAK becomes first detected during late diplotene (Figure 1A and 1B). During this stage, homologues are desynapsing and their LEs separate, and discrete MCAK signals appeared at one of their ends (arrows in Figure 1A). Since mouse chromosomes are telocentric, the centromeric MCAK signals were located at the nuclear periphery, where the LE ends were associated. In some chromocentres, which represent clustered centromere heterochromatic regions of several autosomes, the MCAK signals did not occupy all the chromocentre volume (Figure 1C–1E). The MCAK signals appeared at the end of the SYCP3-labelled LEs, and coalesced between them (Figure 1D). When the centromeric ends of single desynapsed LEs were discerned, the MCAK signal frequently appeared as two closely associated dots, one per sister chromatid (Figure 1F and 1G). During diakinesis, the MCAK labelling at centromeres was similar. Interestingly, in the sex body the MCAK signal corresponding to the Y centromere was brighter and larger than that found at the centromere of the X chromosome (Figure 1H and 1I).

It has been demonstrated that the kinase Aurora-B recruits MCAK to the centromere and phosphorylates it during mitosis, and that this modification regulates the MCAK depolymerising activity [21–23]. To verify if Aurora-B also appears before MCAK at centromeres during meiosis, we performed a double immunolabelling of MCAK and Aurora-B. We have previously described that in mouse spermatocytes Aurora-B is first detected at centromeres during late pachytene, occupying the entire centromeric heterochromatin, but that at late diplotene it relocalises to discrete regions near the kinetochores [27]. Accordingly, our results showed that in early diplotene spermatocytes Aurora-B occupied the centromeric heterochromatic regions, and that MCAK was absent from centromeres (left cell in Figure 1J and 1K). However, MCAK mostly colocalised with Aurora-B to discrete centromere regions during late diplotene (right cell in Figure 1J and 1K). In some homologous centromeres it was evident that both proteins colocalised appearing as two associated spots, one per sister chromatid (inset in Figure 1J). Thus, the appearance of MCAK is coincident with the reorganisation of Aurora-B at late diplotene centromeres. Consequently, Aurora-B appears first at centromeres during meiosis I, and as during mitosis, it probably recruits MCAK.

To accurately localise the centromere domain where MCAK appears we made a double immunolabelling of MCAK and kinetochores by using an anti-centromere autoantibody serum (Figure 1L–1N). In late diplotene spermatocytes, the kinetochore signals appeared slightly displaced from, or in between the two MCAK signals present at each homologous centromere (insets in Figure 1L and 1M). Thus, at least during late diplotene, MCAK and kinetochores occupy different centromeric domains.

### MCAK, SYCP3, and Aurora-B Appear at the Inner Domain of Metaphase I Centromeres but at Different Topological Subdomains

**Figure 2:**
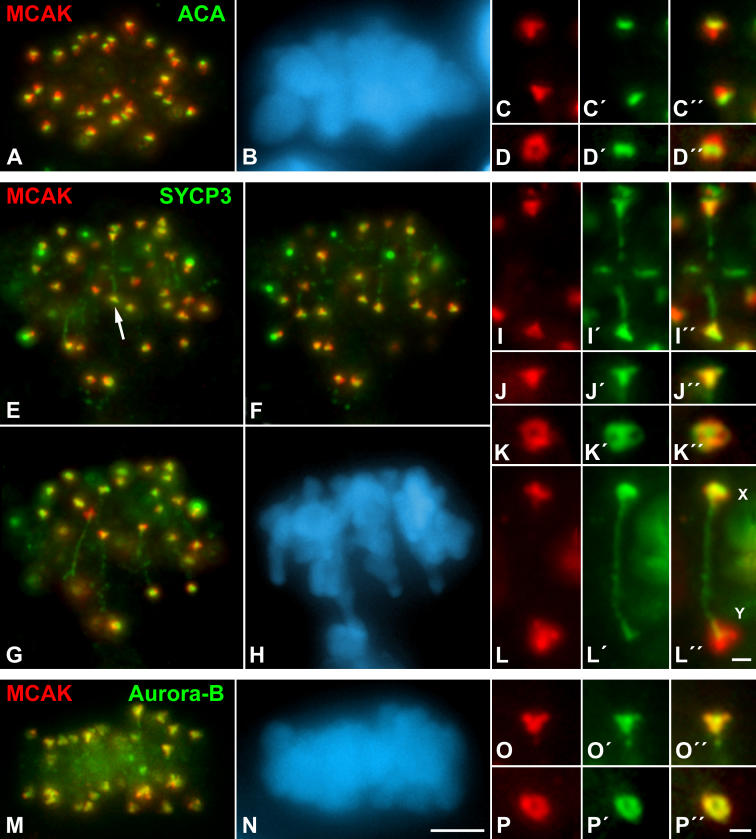
MCAK and Aurora-B Colocalise at the Inner Domain of Metaphase I Centromeres MCAK is labelled in red and kinetochores (A–D″), SYCP3 (E–L″), and Aurora-B (M–P″) are labelled in green. In merged images colocalisation regions appear in yellow. DNA appears in blue. All images are z-projections of several focal planes throughout the metaphase I spermatocytes shown. (A,B**)** Metaphase I. MCAK signals appear below kinetochore ones. (C–C″) Side view of a metaphase I autosomal bivalent. MCAK is detected as T-like signals below, and partially colocalising with, kinetochore signals in both homologues. (D–D″) Top view of an autosomal metaphase I centromere. MCAK is detected as a ring that surrounds the closely associated sister kinetochores. (E–H) Three projections of different focal planes of a metaphase I spermatocyte. MCAK colocalises with SYCP3 signals at centromeres. (I–I″) Side view of the autosomal bivalent arrowed in E. MCAK signals mostly colocalise with SYCP3 signals at centromeres. Note the labelling of SYCP3 at the interchromatid domain. (J–J″) Side view of an autosomal metaphase I centromere. MCAK and SYCP3 mostly colocalise. (K–K″) Top view of an autosomal metaphase I centromere. While MCAK is detected as a single ring, the SYCP3 signal appears as two closely associated rings. (L–L″) Side view of a metaphase I sex bivalent. The MCAK and SYCP3 signals are very similar at the X centromere. However, MCAK stains intensely the Y centromere while SYCP3 is barely visible. The interchromatid domain is labelled with SYCP3, but not with MCAK. (M,N) Metaphase I. MCAK and Aurora-B colocalise at centromeres. (O–O″) Side view of an autosomal metaphase I centromere. MCAK and Aurora-B colocalise at the inner centromere domain showing a T-like appearance. (P–P″) Top view of an autosomal metaphase I centromere. MCAK and Aurora-B colocalise as a single ring. Bars, 5 μm (A,B,E–H,M,N), 0.5 μm (C–C″,I–I″,L–L″), and 1 μm (D–D″,J–K″,O–P″).

In prometaphase I and metaphase I spermatocytes (Figure 2), the MCAK staining appeared at centromeres partially colocalising with kinetochore signals (Figure 2A and 2B). A careful inspection of side-viewed centromeres, showed that MCAK appeared as T-shaped signals below the closely associated sister kinetochores, but showing some degree of colocalisation with them (Figure 2C–2C″). Thus, MCAK appears at the inner centromere domain. Surprisingly, when centromeres were top-viewed, a single MCAK ring was discerned surrounding the sister kinetochores (Figure 2D–2D″).

Since we have previously described the presence of SYCP3 at the inner domain of metaphase I centromeres [28], we performed a double immunolabelling of MCAK and SYCP3 to test if these proteins colocalised. In metaphase I spermatocytes, these two proteins colocalised at centromeres, but not at the interchromatid domain where only SYCP3 was present (Figure 2E–2H). When their distribution in autosomal bivalents was observed at higher magnification it was evident that the MCAK signals colocalised but were slightly thicker than the SYCP3 ones when centromeres were side-viewed (Figure 2I–2I″ and 2J–2J″). Remarkably, when centromeres were top-viewed MCAK appeared as a single ring, but the SYCP3 signal was composed by two closely associated rings that partially colocalised with MCAK (Figure 2K–2K″). Interestingly, the labelling of MCAK and SYCP3 at the centromeres of the sex chromosomes was different. The signals for both proteins mostly colocalised at the centromere of the X chromosome (Figure 2L–2L″), as in the autosomal centromeres. However, the centromere of the Y chromosome showed a very large and bright T-shaped MCAK signal while the SYCP3 signal was smaller (Figure 2L–2L″).

Since we have previously described the presence of Aurora-B at the inner centromere domain [27], we also analysed the relative distribution of MCAK and Aurora-B at metaphase I centromeres. Our results showed that both proteins colocalised at the inner centromere domain (Figure 2M and 2N) appearing as T-like signals when centromeres were side-viewed (Figure 2O–2O″) and as single rings when centromeres were top-viewed (Figure 2P–2P″). Thus, although MCAK, Aurora-B, and SYCP3 are present at the inner domain, they occupy different topological subdomains. The finding that the MT-depolymerising kinesin MCAK and Aurora-B form a ring below the closely associated sister kinetochores is particularly relevant and intriguing.

### MCAK Is Not Detected at Centromeres from Telophase I to Late Interkinesis

**Figure 3:**
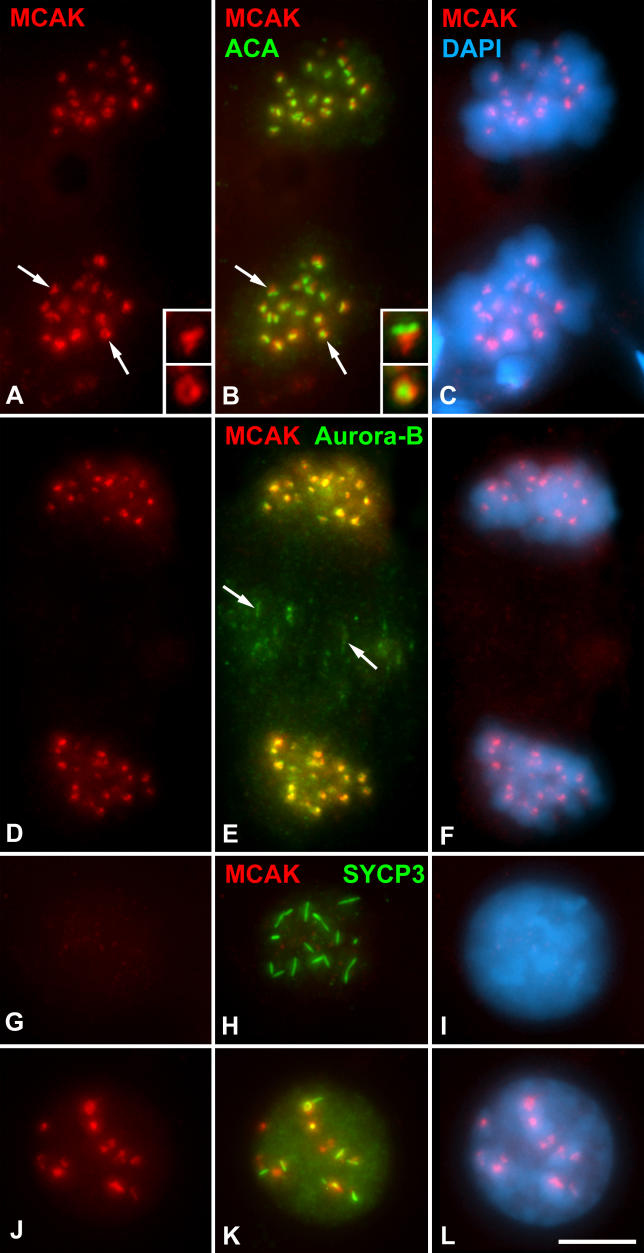
MCAK Is Lost from Centromeres at Telophase I but Is Again Detected at Centromeres by Late Interkinesis MCAK is labelled in red and kinetochores (B), Aurora-B (E), and SYCP3 (H,K) are labelled in green. In merged images colocalisation regions appear in yellow. DNA appears in blue. All images are z-projections of several focal planes throughout the spermatocytes shown. (A–C) Anaphase I. The MCAK labelling is detected in side views of the centromere as a T-shaped signal below the closely associated sister kinetochores (top insets), or as a ring surrounding them in top views of the centromere (bottom insets). Arrows in (A) and (B) indicate the enlarged centromeres shown in insets. (D–F) Late anaphase I. MCAK and Aurora-B signals at centromeres colocalise. Aurora-B is also observed at the spindle midzone (arrows). (G–I) Early interkinesis nucleus. SYCP3 appears as thin bars in the nucleoplasm, while no MCAK labelling can be detected. (J–L) Late interkinesis nucleus. SYCP3 bars are still visible in the nucleus, and MCAK is again detected as small patches on heterochromatic chromocentres. Bar, 10 μm.

During anaphase I (Figure 3), MCAK signals were evident at centromeres where they appeared, as in metaphase I, as a T-shaped structures below the closely associated sister kinetochores at the inner centromere domain (Figure 3A–3C). Interestingly, top-views of the centromeres showed that the MCAK ring signal was still surrounding both sister kinetochores (inset in Figure 3A and 3B). During late anaphase I, MCAK colocalised with Aurora-B at centromeres (Figure 3D–3F), but not at the spindle midzone, where Aurora-B was present along some overlap MTs (arrows in Figure 3E). During early telophase I, the MCAK signals became less intense and smaller than during anaphase I, and disappeared by late telophase I. In early interkinesis nuclei, an interphase-like stage between both meiotic divisions but without DNA replication, remnants of SYCP3 appeared as elongated bars on chromocentres, but MCAK labelling was not detected (Figure 3G–3I). During this stage, Aurora-B reappeared at chromocentres (unpublished data). However, by late interkinesis, when SYCP3 was still visible as shorter bars, MCAK was again detected at centromeres as round agglomerates (Figure 3J–3L), that colocalised with Aurora-B (unpublished data). These results show that although MCAK, Aurora-B, and SYCP3 are present at the inner domain of metaphase I centromeres, their behaviours are completely different after anaphase I, and as during prophase I, Aurora-B appears before MCAK during interkinesis.

### MCAK Redistributes within Centromeres throughout Congression to the Metaphase II Plate to Appear as a Perikinetochoric Ring

**Figure 4:**
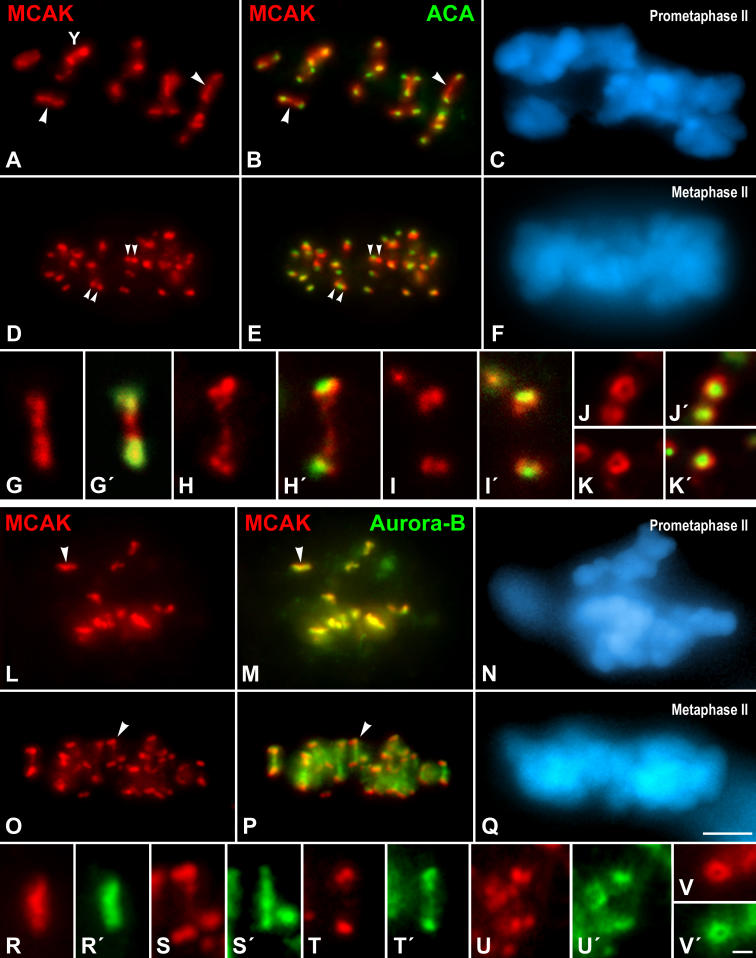
MCAK and Aurora-B Relocalise at Centromeres during the Prometaphase II/Metaphase II Transition MCAK is labelled in red and kinetochores (A–K′) and Aurora-B (L–V′) are labelled in green. In merged images colocalisation regions appear in yellow. DNA appears in blue. All images are z-projections of several focal planes throughout the spermatocytes shown. (A–C) Prometaphase II. MCAK appears as a band traversing the centromere (arrowheads) and joining sister kinetochores. The signal at the Y centromere (Y) is brighter than the autosomal ones. (D–F) Metaphase II. MCAK is detected as pairs of dots (double arrowheads) below each sister kinetochore. (G–I′) Three centromeres in prometaphase II/metaphase II sequentially placed from G to I′. In prometaphase II MCAK appears as a band that crosses the centromere and joins sister kinetochores (G,G′). With ongoing chromosome congression MCAK progressively disappears from the inner centromere domain to accumulate as a pair of dots below each sister kinetochore (H,H′). When all chromosomes are aligned at the metaphase II plate, MCAK is only detected as two dots under each sister kinetochore (I,I′). (J–K′) Top views of two metaphase II kinetochores. Each sister kinetochore is encircled by an MCAK ring. (L–N) Prometaphase II. MCAK colocalises with Aurora-B at a band that traverses the centromere. The centromere indicated in Q (arrowhead) has been enlarged in R,S. (U–X) Metaphase II. The centromere indicated in L,M (arrowhead) has been enlarged in R,R′. (O–Q) Metaphase II. MCAK appears as two pairs of dots at each centromere, while Aurora-B is still present as a band that traverses the centromere. The centromere indicated in O,P (arrowhead) has been enlarged in S,S′. (T,T′) Side view of a metaphase II centromere. MCAK is visible as two pairs of dots, whereas Aurora-B begins to concentrate at kinetochores but is still present at the inner domain. (U,U′) Side view of a late metaphase II centromere. MCAK and Aurora-B colocalise as pairs of dots. (V,V′) Top view of a metaphase II kinetochore. MCAK and Aurora-B colocalise as a ring. Bars, 2.5 μm (A–F,L–Q) and 0.5 μm (G–K′,R–V′).

MCAK was clearly detected at early prometaphase II centromeres (Figure 4) as a rough band that traversed the entire centromere and joined sister kinetochores (Figure 4A–4C). These signals redistributed below both sister kinetochores when all chromosomes were aligned at the metaphase II plate (Figure 4D–4F). Accordingly, we found different morphologies of MCAK signals between prometaphase II and metaphase II. In early prometaphase II, all chromosomes showed an MCAK band that crossed the centromere and partially colocalised with the kinetochore signals (Figure 4A–4C, 4G, and 4G′). Then, during chromosome congression, this band started to disappear becoming more diffuse. Concomitantly, MCAK accumulated as two spots below each sister kinetochore (Figure 4H and 4H′). It must be stressed that this redistribution of MCAK did not affect to all chromosomes simultaneously. Therefore, different prometaphase II chromosomes in a given spermatocyte could show different morphologies of MCAK signals at their centromeres. By metaphase II, however, most chromosomes showed pairs of MCAK dots below each sister kinetochore (Figure 4D–4F, 4I, and 4I′). Whenever the centromeres of aligned metaphase II chromosomes were top-viewed, a single MCAK ring was observed around each kinetochore (Figure 4J–4K′). Taking into account the observations of side- and top-viewed metaphase II centromeres, it is clear that MCAK forms a ring below individual kinetochores. These MCAK rings presented an average diameter of 0.63 ± 0.05 μm (*n* = 30), while their inner hole had an average diameter of 0.27 ± 0.03 μm (*n* = 30). To test whether the redistribution of MCAK depends on its phosphorylation status we used an antibody that recognises phosphorylated MCAK on Ser92 [21], but it did not work, even when microcystine, an inhibitor of phosphatases, was used in the fixative solution.

We also measured the interkinetochore distance in chromosomes when MCAK appeared either as a band or as two spots below each kinetochore. When MCAK was detected as a band, the average interkinetochore distance was of 1.10 ± 0.12 μm (*n* = 15), while when MCAK was localised as a ring below kinetochores, the average interkinetochore distance was 1.93 ± 0.40 μm (*n* = 32). Thus, the interkinetochore distance was increased by 75% on chromosomes where MCAK was visualised as a perikinetochoric ring. This centromere stretch suggests that tension across centromeres generated during chromosome congression influences the distribution of MCAK.

### MCAK and Aurora-B Redistribute as a Perikinetochoric Ring at Different Times during Chromosome Congression to the Metaphase II Plate

We have previously described that Aurora-B is detected as a connecting strand in between sister kinetochores in metaphase II spermatocytes [27]. To test whether the redistribution of MCAK during chromosome congression was accompanied by a similar relocalisation of Aurora-B, we made a double immunolabelling of these proteins. Our results showed that MCAK and Aurora-B colocalised at a connecting strand in every prometaphase II centromere (Figure 4L–4N, 4R, and 4R′). However, as chromosomes congressed to the metaphase II plate, MCAK redistributed below kinetochores as pairs of dots while Aurora-B still persisted as a connecting strand between sister kinetochores (Figure 4O–4Q, 4S, and 4S′). Posteriorly, at late metaphase II, this Aurora-B strand became diffuse (Figure 4T and 4T′) and concentrated as a ring below kinetochores that colocalised with the MCAK one (Figure 4U–4V′). Thus, both proteins concentrate as a perikinetochoric ring but with a different timing during chromosome alignment to the metaphase II plate.

### MCAK Relocalisation at Centromeres during Chromosome Congression to the Metaphase II Plate Depends on MT Attachment to Kinetochores

**Figure 5:**
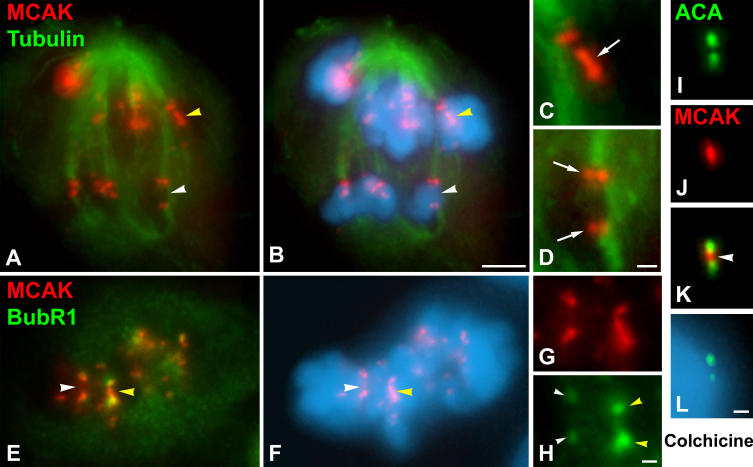
MCAK Reorganisation at Metaphase II Centromeres Depends on MT Attachment or Tension across the Centromere MCAK is labelled in red, α-tubulin (A–D), BubR1 (E–H), and kinetochores (I–L) in green, and DNA in blue. (A and B) Prometaphase II. MCAK appears either as a band that traverses the centromere (yellow arrowhead) when they are not associated with bundles of kMTs, or as two pairs of dots (white arrowhead) when centromeres are associated with bundles of kMTs from opposite poles. (C and D) Enlargements of the centromeres indicated in (A) and (B). Arrow in (C) indicates the band of MCAK, and arrows in (D) the pairs of MCAK signals. (E and F) Prometaphase II. When MCAK appears as a band (yellow arrowhead) BubR1 shows an intense labelling at kinetochores, while when MCAK is observed as pairs of dots (white arrowhead) the BubR1 signals at sister kinetochores are faint. (G and H) Enlargements of the centromeres indicated in (E) and (F). Yellow arrowheads in (H) indicate intense BubR1 signals, and white arrowheads in (H) faint BubR1 signals. (I–L) Selected centromere from a colchicine-treated metaphase II spermatocyte. MCAK appears as a small patch (arrowhead in [K]) in between sister kinetochores. Bars, 2.5 μm (A,B,E,F) and 0.5 μm (C,D,G,H,I–L).

The change of localisation of MCAK that we have observed at prometaphase II centromeres is similar to its redistribution at mitotic centromeres, which is dependent on the attachment of MTs to kinetochores [21]. To corroborate this hypothesis we made a double immunolabelling of MCAK and spindle MTs labelled with an anti-α-tubulin antibody (Figure 5). To test if the change of MCAK localisation depends on MT attachment to kinetochores we looked for prometaphase II spermatocytes in which some centromeres presented an MCAK band traversing centromeres, and other ones with double spot signals (Figure 5A and 5B). We found that those chromosomes that had an MCAK band at their centromeres were not attached to kMTs (Figure 5C). By contrast, bundles of kMTs from opposite poles reached sister kinetochores at centromeres where MCAK was present as pairs of dots below each kinetochore (Figure 5D).

We next performed a double immunolabelling of MCAK and the checkpoint protein BubR1 to test the relationship between MT attachment or tension across prometaphase II centromeres and the morphology of the MCAK signals. BubR1 kinase is an essential component of the spindle checkpoint machinery that localises to unattached kinetochores, and is mostly released from kinetochores once kMTs have stably attached [29,30]. Our results showed that whenever MCAK appeared as a band traversing the centromere BubR1 intensely labelled the kinetochores, while when MCAK was observed as pairs of dots below kinetochores they showed a very faint BubR1 labelling (Figure 5E–5H). Moreover, after treatment with colchicine, an MT-depolymerising drug, MCAK was never detected as pairs of dots, but as a small patch between sister kinetochores (Figure 5I–5L). We noticed that, due to the use of colchicine, sister kinetochores were closer between them than in non-treated chromosomes. Altogether, these results support that the MCAK redistribution at prometaphase II centromeres depends on MT attachment to kinetochores creating tension across centromeres.

### A Connecting Strand Is Ultrastructurally Revealed at Metaphase II Centromeres

**Figure 6:**
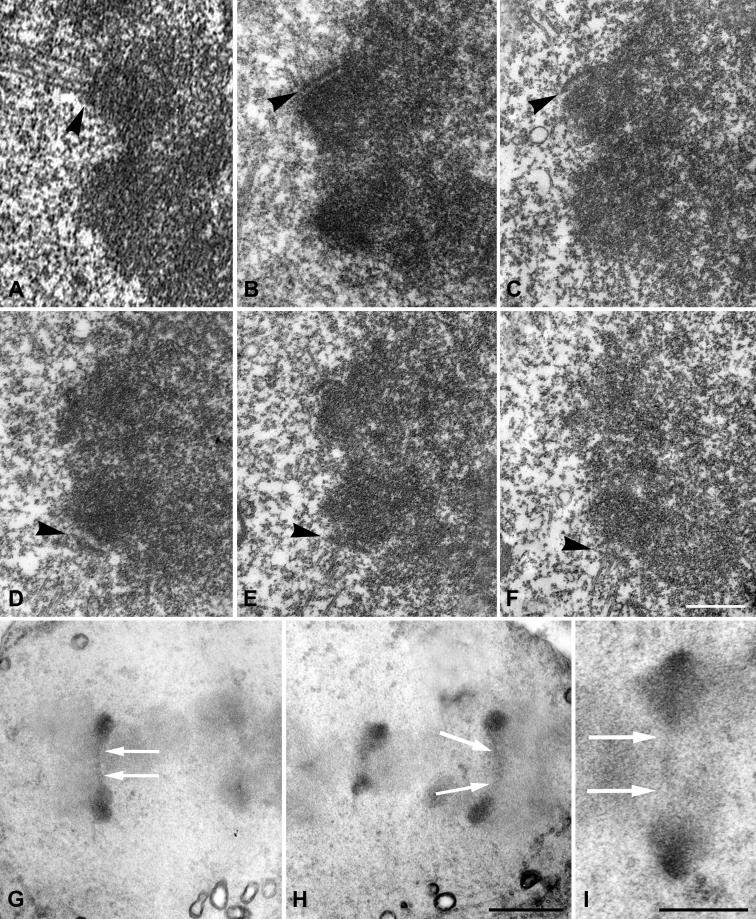
Ultrastructure of Metaphase II Centromeres (A–F) Six serial sections of a metaphase II centromere. Conventional technique. The outer plates of both sister kinetochores (arrowheads) are clearly differentiated. (G and H) Two sections of metaphase II centromeres after the Os-PPD technique. The condensed chromosomes show a very low contrast, while sister kinetochores show a high contrast. The kinetochores appear joined by a continuous band of medium contrast material (arrows). (I) Centromeric region of a chromosome in late metaphase II. A low contrast material extends as a thin and discontinuous band (arrows) joining the highly contrasted sister kinetochores. Bars, 0.5 μm (A–F), 2.5 μm (G,H), and 2.5 μm (I).

Since MCAK appeared as a band traversing the centromere and joining sister kinetochores, as it occurs for the chromosomal passenger proteins Aurora-B and INCENP [27], we tested whether a differentiated structure could be ultrastructurally revealed at prometaphase and metaphase II centromeres. We tried to reveal this connecting strand by using a conventional technique for electron microscopy (Figure 6). In serial sections we discerned the two separated trilaminar sister kinetochores facing opposite poles, particularly the outer kinetochore plates (arrowheads in Figure 6A–6F). However, we did not observe signs of any connecting strand with a differential contrast between sister kinetochores. We also employed the Os-PPD cytochemical technique that preferentially detects ribonucleoproteins [31,32], and that in mouse metaphase I centromeres reveals the inner domain [28]. With this technique the condensed prometaphase II/metaphase II chromosomes presented a low contrast, while the centromeres appeared highly contrasted (Figure 6G). A detailed observation of centromeres showed two contrasted round structures corresponding to sister kinetochores and the underlying chromatin (Figure 6G and 6H). Additionally, a dense strand between sister kinetochores was also observed (arrows in Figure 6G and 6H). At the onset of anaphase II, the material conforming this connecting strand between sister kinetochores showed a lower contrast and became diffuse, while the staining beneath each sister kinetochore increased (Figure 6I). This result demonstrates that the inner domain of metaphase II centromeres has a protein/ribonucleoprotein composition different to that found in the condensed chromatin at the arms.

## Discussion

### The Mechanisms Underlying the Inner Centromere Domain Assembly Might Be Conserved during Mitosis and Meiosis

We have found that MCAK loads at centromeres after Aurora-B during late diplotene and late interkinesis. Recently, it has been reported that Aurora-B is required for the targeting of MCAK at mitotic centromeres, and that Aurora-B phosphorylates MCAK and regulates its activity [21–23]. Thus, our results support those obtained in somatic cells and suggest that Aurora-B kinase is essential for the MCAK loading in both somatic cells and spermatocytes. Moreover, we have previously reported that Aurora-B was loaded at meiotic centromeres after the chromosomal passenger protein INCENP [27]. This same sequence of loading at centromeres has also been reported for INCENP and Aurora-B in somatic cells [33,34]. Altogether, all these data strongly support that the mechanisms underlying the inner centromere domain assembly, regarding the loading of chromosomal passenger proteins and MCAK, are conserved in vertebrates during both mitosis and male meiosis.

### The Inner Domain of Metaphase I Centromeres

**Figure 7:**
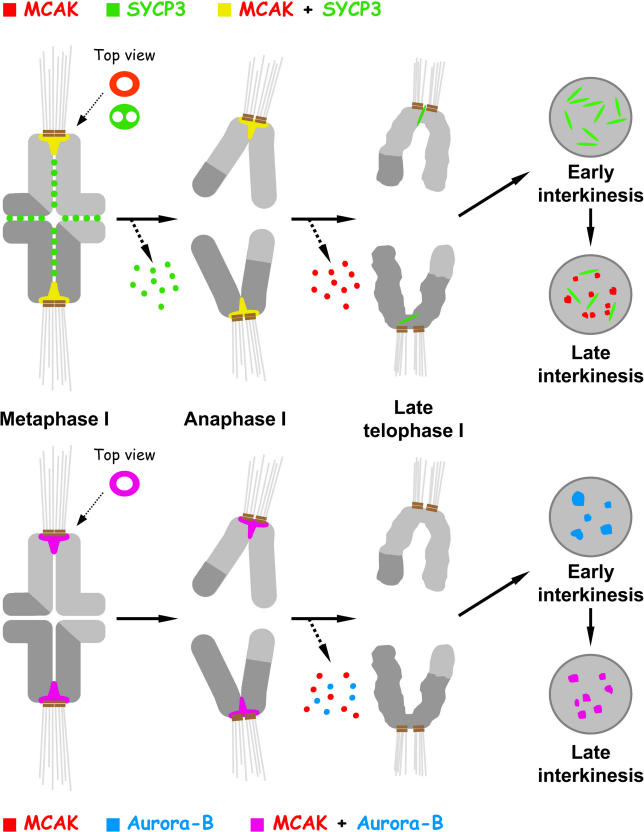
Schematic Representation of the Distribution of MCAK, SYCP3, and Aurora-B from Metaphase I to Late Interkinesis One homologue is depicted in light grey and another in darker grey. Trilaminar kinetochores are indicated in brown, kMTs in light grey, MCAK in red, SYCP3 in green, and Aurora-B in blue. In metaphase I bivalents and segregating anaphase I chromosomes, MCAK, SYCP3, and Aurora-B colocalise at the inner centromeric domain below the closely associated sister kinetochores when centromeres are side-viewed. In top-viewed centromeres, MCAK, and Aurora-B appear as a single ring while SYCP3 is present as two closely associated rings. The SYCP3 labelling at the interchromatid domain in metaphase I bivalents disappears during anaphase I. At late telophase I, MCAK, and Aurora-B have been lost from centromeres, while SYCP3 rearranges as bars that may appear either in between individualised sister kinetochores or separated from them. In early interkinesis nuclei, SYCP3 appears as bars, and Aurora-B appears as large patches colocalising with chromocentres. In late interkinesis nuclei, SYCP3 still persists as bars while MCAK reappears at chromocentres and colocalises with Aurora-B.

Our results show that MCAK is detected as a spot in late diplotene centromeres, and then accumulates as a T-shaped signal below the closely associated sister kinetochores during metaphase I. Similar T-shaped signals at the inner domain of metaphase I centromeres have been previously described in mouse spermatocytes by immunofluorescence for the chromosomal passenger proteins INCENP and Aurora-B [27], the cohesin subunit radiant sensitive protein 21(RAD21), and the LE proteins SYCP3 and SYCP2 [28]. Likewise, this T-shaped domain has also been detected by electron microscopy by silver staining [35,36] and the Os-PPD cytochemical technique [28]. At first sight, it seems that all these proteins mostly colocalise at the inner domain of metaphase I centromeres. However, this is only observed when centromeres are side-viewed. The observation of top-views of metaphase I centromeres reveals that MCAK and Aurora-B appear as a single ring surrounding sister kinetochores, thus corresponding to a “cone”-like three-dimensional distribution (Figure 7). By contrast, SYCP3, SYCP2, and RAD21 appear as a double-ring, being consistent with a “double cornet”-like arrangement [28]. Thus, taken together our results demonstrate the existence of distinct topological subdomains within the inner domain of metaphase I centromeres, at least in male mouse. This proposal is supported by the patterns of distribution of MCAK, INCENP, Aurora-B, RAD21, SYCP3, and SYCP2 at the centromere of the Y chromosome. While MCAK, Aurora-B, and INCENP [27] show an intense T-shaped labelling, SYCP3, SYCP2, and RAD21 appear as smaller and fainter signals below sister kinetochores. We envisage that the differential distribution of particular centromeric DNA satellite sequences [37] could lead to the preferential association of distinct inner centromere proteins.

### MCAK Function at Meiosis I Centromeres

In mitosis, MCAK has been involved in eliminating improper merotelic orientations that appear when a single kinetochore becomes attached to MTs from opposite spindle poles rather than just to one pole [20]. These merotelic kinetochore orientations are relatively frequent during early mitosis in mammalian somatic cells probably due to the proximity between sister kinetochores [38]. However, during the first meiotic division the homologous centromeres, each one with two closely associated sister kinetochores, i.e. with a single functional kinetochore, are far apart, and consequently the expected frequency of merotelic orientations may be lower. Nevertheless, it cannot be ruled out that MCAK could also be involved in eliminating these improper kinetochore-MT interactions during meiosis I. In this sense, the “cone”-like appearance of MCAK below the closely associated sister kinetochores at metaphase I centromeres is surprising taking into account its MT-depolymerising activity, since only the ring surrounding the sister kinetochores would be accessible to spindle MTs. This perikinetochoric ring of MCAK could break aberrant MT connections around the two sister kinetochores. Therefore, it is possible that MCAK could assist in setting up the mono-orientation of the two sister kinetochores by permitting strong MT binding in the centre of the sister kinetochores, and then trimming MTs from the sides of the centromere. This would produce a bundle from which rogue MTs would not escape.

Since in mitosis Aurora-B regulates the MCAK activity by phosphorylating it [21–23], and these two proteins are located at the same topological subdomains at the inner domain of metaphase I centromeres, it is tempting to suggest that Aurora-B could also regulate the activity of MCAK during the first meiotic division. Nevertheless, contrary to what happens in mitosis, a relocalisation of MCAK that occurs when it is dephosphorylated probably by phosphatase 1 [21], is not detected at metaphase I centromeres. In this context, it is worth mentioning that the pulling forces exerted by bundles of kMTs from opposite poles on the homologous centromeres do not create tension across centromeres as during mitosis or metaphase II. By contrast, the generated tension is supported by the chromosome arms where cohesin complexes ensure the bivalent integrity until the onset of anaphase I. This situation could explain why we did not observe a redistribution of MCAK at metaphase I centromeres.

Our findings show that MCAK persists as a “cone”-like signal at centromeres during anaphase I. This persistence suggests that MCAK may participate in the depolymerisation and shortening of kMTs during homologue segregation. In this respect, it has been proposed that MCAK could also be involved in anaphase chromosome segregation in somatic cells [19]. Alternatively, MCAK may persist at centromeres without any significant function.

We have found that although MCAK disappears from centromeres during telophase I, it is again detectable at centromeres by late interkinesis (Figure 7). This is not surprising since there are other proteins that show a similar pattern of disappearance at telophase I and reappearance at interkinesis/prometaphase II, for instance the motor protein CENP-E [39], Aurora-B and INCENP [27], Borealin, and also several checkpoint proteins like BubR1 and Mad2 (unpublished data). Thus, MCAK, as any of these other mentioned proteins, may diffuse to the cytoplasm or nucleoplasm during chromosome decondensation at telophase I, to be then recruited to the centromeres by late interkinesis. Nevertheless, this possibility seems unlikely since we have never observed an increase in the MCAK fluorescence at the nucleoplasm or cytoplasm during telophase I and early interkinesis. Therefore, although we have no direct proof, we currently favour the idea that MCAK is degraded at telophase I, resynthesised in early interkinesis and then recruited to centromeres by Aurora-B during late interkinesis.

### The Inner Domain of Metaphase II Centromeres

We have found that when prometaphase II centromeres are not attached to kMTs MCAK appears at the inner domain as a connecting strand between sister kinetochores. We have previously observed a similar pattern of labelling for INCENP and Aurora-B [27]. These stainings are reminiscent of those previously obtained by silver staining in grasshopper metaphase II chromosomes [40,41]. In those studies this structure was named “connecting strand” by its similarity with the labelling obtained in mitotic chromosomes with antibodies recognising the CLiP proteins [4]. During last years, a similar connecting strand was found at the inner centromere domain of mitotic chromosomes for MCAK [11] and DNA topoisomerase IIα [42]. Our ultrastructural observations of conventionally contrasted sections do not reveal any differentiation between the sister kinetochores at metaphase II centromeres. By contrast, we have detected after the Os-PPD technique a differentially contrasted connecting strand between sister kinetochores. The inner domain of metaphase I centromeres is also revealed with the Os-PPD technique [28]. These results suggest the existence of distinct proteins, probably ribonucleoproteins [31], or a differential chromatin conformation at the inner domain of both metaphase I and II centromeres.

### MCAK Function at Meiosis II Centromeres

**Figure 8:**
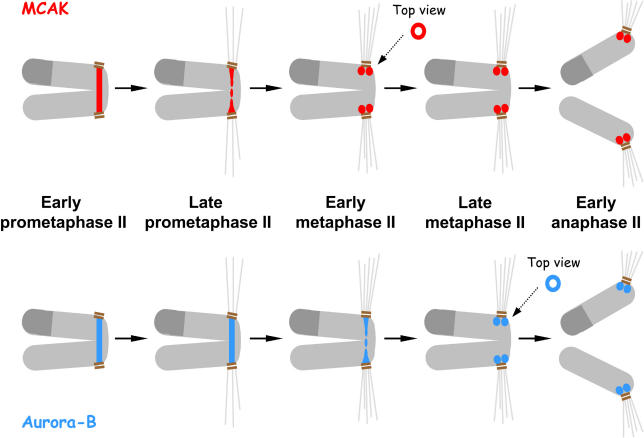
Schematic Representation of the Distribution of MCAK and Aurora-B from Early Prometaphase II to Early Anaphase II The chromosome is depicted with one chromatid in light grey, and the sister one both in light and darker grey to indicate that it recombined. Trilaminar kinetochores are indicated in brown, kMTs in light grey, MCAK in red, and Aurora-B in blue. In early prometaphase II, MCAK and Aurora-B colocalise as a band at the inner centromeric domain between sister kinetochores. Once chromosomes attach to kMTs from opposite poles, MCAK relocalises to appear as a pair of spots below both kinetochores at early metaphase II. In these top-viewed centromeres MCAK appears as a ring that encircles kinetochores. In turn, Aurora-B also relocalises as a pair of spots below kinetochores but at late metaphase II, where as MCAK, it encircles kinetochores. The MCAK and Aurora-B perikinetochoric rings are still evident during early anaphase II.

Our results show that MCAK and Aurora-B appear as a band that traverses the centromere during prometaphase II when kMTs are not attached to sister kinetochores, and consequently the spindle checkpoint machinery is activated, as revealed by the strong presence of BubR1 at those kinetochores. However, during chromosome congression to the metaphase II plate, MCAK redistributes below each sister kinetochore appearing as a ring, while Aurora-B still persists as a band traversing the centromere until late metaphase II (Figure 8). A similar redistribution of MCAK at centromeres occurs during chromosome congression in mitosis [21]. In this sense, it has been proposed that phosphatase 1 could dephosphorylate MCAK at the inner domain of mitotic chromosomes and promote its redistribution [21].

Recently, it has been proposed that during prometaphase, when MCAK is located at the inner domain of mitotic centromeres, its MT-depolymerising activity would be decreased by Aurora-B phosphorylation [21]. In this context, it can be speculated that MCAK would not have any role when present at the inner domain. Nevertheless, it has been recently demonstrated that ICIS, a new protein localised in an MCAK-dependent manner to the inner centromere domain, forms a complex with MCAK, and stimulates its MT-depolymerising activity in vitro [26]. It has been proposed that the complex MCAK-ICIS destabilises MTs that are laterally associated with inner centromeres thus preventing erroneous merotelic attachments. Moreover, since ICIS also interacts with the passenger proteins INCENP and Aurora-B, it has been suggested that ICIS promotes the correct chromosome biorientation [26]. In our opinion, the MCAK-ICIS mediated mechanism to avoid kinetochore-MT attachment errors would be specially relevant in mouse chromosomes since they are telocentric, and then the inner centromere domain would be more accessible to MTs than in submetacentric or metacentric chromosomes. Thus, the role of MCAK at the inner centromere domain of mouse prometaphase II chromosomes could be the same to that hypothesised for MCAK in mitosis, i.e. to prevent incorrect merotelic attachments.

Our results show that MCAK and Aurora-B appear as a perikinetochoric ring in aligned metaphase II chromosomes. Which MTs do MCAK depolymerise and why at this perikinetochoric domain? We envision that MCAK could depolymerise MTs attached at the centromeric chromatin surrounding kinetochores. This would define the identity of bundles of kMTs, making easier the biorientation of bivalents and chromosomes during metaphase I and metaphase II, respectively. Alternatively, MCAK could depolymerise kMTs during chromatid segregation. In fact, antisense and dominant-negative experiments suggested a possible function of MCAK not only in chromosome alignment, but also in poleward movement of chromatids during mitotic anaphase [19].

### MCAK and Aurora-B Define a Novel Transient Centromere Domain in Mouse Meiotic Chromosomes

We have detected for the first time that MCAK and Aurora-B are redistributed under the sister kinetochores at metaphase II centromeres appearing as a perikinetochoric ring. This labelling resembles those observed for MCAK and Aurora-B in top-views of metaphase I centromeres. We propose that those perikinetochoric rings could represent a novel centromere domain, at least in mouse chromosomes during male meiosis, and that the peculiar conformation of this domain could be determined by the particular distribution of different satellite DNAs at mouse centromeres [37]. However, it must be stressed that the perikinetochoric ring is a transient centromere domain since, at least during meiosis II, it is only discerned when poleward pulling forces exerted by kMTs from opposite poles create tension across centromeres. MCAK and Aurora-B are not the only proteins that can be detected at these perikinetochoric rings. We have detected that SGO2, a member of a protein family known as shugoshin that protect centromeric cohesin complexes from separase cleavage during anaphase I [43,44], appears as a perikinetochoric ring at centromeres of aligned mouse metaphase II and metaphase mitotic chromosomes (unpublished data). Besides that function, SGO2 may also be implied in the tension-sensing machinery, since it presents a potential MT binding region similar to SGO1, another protein of this family that has been related with this process [45]. Thus, it is certainly tempting to propose that the perikinetochoric ring is a transient centromere domain that appears when tension across centromeres is generated, and whose function is to correct improper MT attachments to kinetochores. Obviously, further studies are needed to corroborate the presence of this perikinetochoric ring in other species during both mitosis and meiosis, and to know the molecular mechanisms regulating its appearance and precise function.

## Materials and Methods

### Squashing of spermatocytes and immunofluorescence microscopy.

Testes from adult male C57BL/6 mice were used. In some experiments, mice weighing approximately 30 g, were intraperitoneally injected with 40 mg/kg of colchicine (Sigma, St. Louis, Missouri, United States) in PBS and sacrificed at 3 h. Testes were removed, detunicated and fixed for squashing and subsequent immunofluorescence as previously described [39,46]. Seminiferous tubules were briefly fixed in freshly prepared 2% formaldehyde in PBS (137 mM NaCl, 2.7 mM KCl, 10.1 mM Na_2_HPO_4_, 1.7 mM KH_2_PO_4_, [pH 7.4]) containing 0.05% Triton X-100 (Sigma). After 5 min, several seminiferous tubules fragments were placed on a slide coated with 1 mg/ml poly-L-lysine (Sigma) with a small drop of fixative, and the tubules were gently minced with tweezers. The tubules were then squashed and the coverslip removed after freezing in liquid nitrogen. The slides were later rinsed three times for 5 min in PBS, and incubated for 45 min at room temperature with primary antibodies. In double-labelling experiments, primary antibodies were incubated simultaneously. Following three washes in PBS, the slides were incubated for 30 min at room temperature with secondary antibodies. The slides were subsequently rinsed in PBS and counterstained for 3 min with 5 μg/ml DAPI (4′,6-diamidino-2-phenylindole). After a final rinse in PBS, the slides were mounted with Vectashield (Vector Laboratories, Burlingame, California, United States) and sealed with nail varnish.

Immunofluorescence image stacks were collected on an Olympus BX61 microscope equipped with epifluorescence optics, a motorised z-drive, and an Olympus DP70 digital camera controlled by analySIS software (Soft Imaging System, Olympus, Hamburg, Germany). Stacks were analysed and processed using the public domain ImageJ software (National Institutes of Health, United States; http://rsb.info.nih.gov/ij). Final images were processed with Adobe Photoshop 7.0 software.

### Antibodies.

To detect MCAK we used affinity-purified sheep and rabbit polyclonal antibodies against human MCAK [19,21] at 1:40 and 1:200 dilutions, respectively. To detect phosphorylated MCAK we used a sheep antibody specifically recognizing MCAK phosphorylated on Ser92 [21]. Kinetochores were detected with a purified human anti-centromere autoantibody (Antibodies Incorporated, Davis, California, United States) at a 1:100 dilution in PBS. To detect SYCP3 we used a guinea pig polyclonal serum against a 12-mer peptide corresponding to residues 27–38 of rat SYCP3 (kindly provided by Dr. Ricardo Benavente; [47]), at a 1:100 dilution in PBS. To detect Aurora-B kinase we employed the mouse monoclonal AIM-1 antibody (Transduction Labs, Franklin Lakes, New Jersey, United States) at a 1:30 dilution. To detect α-tubulin we employed a mouse monoclonal anti-α-tubulin antibody (Sigma T-5168) at a 1:1,000 dilution. An affinity purified sheep polyclonal antibody against human BubR1 (SBR1.1, kindly provided by Dr. Stephen S. Taylor, [48]), was used at a 1:50 dilution. The secondary antibodies used were a combination of Texas Red-conjugated donkey anti-sheep IgG (Jackson, West Grove, Pennsylvania, United States) at a 1:40 dilution, with either fluorescein isothiocyanate (FITC)-conjugated goat anti-human IgG (Jackson) at a 1:150 dilution, a FITC-conjugated donkey anti-guinea pig IgG (Jackson) at a 1:150 dilution, or a FITC-conjugated goat anti-mouse IgG (Jackson) at a 1:150 dilution.

### Transmission electron microscopy.

Seminiferous tubules were processed for electron microscopy as previously described [49]. Briefly, seminiferous tubules were fixed in 2.5% glutaraldehyde in phosphate buffer (pH 7.2) at room temperature for 5 min. Then, 3 volumes of 8% tannic acid (Merck, Darmstadt, Germany) in phosphate buffer were added for 1 h. Fixed seminiferous tubules were washed in phosphate buffer, and postfixed in 2% osmium tetroxide at room temperature for 1 h. Finally, fixed tubules were washed in phosphate buffer, dehydrated in an ethanol series, and embedded in Epon 812. Ultrathin sections were obtained in a Reichert-Jung ultramicrotome, transferred to copper/rhodium grids, contrasted with uranyl acetate and lead citrate, and observed.

Mouse testes were subjected to the osmium tetroxide/p-phenylenediamine (Os-PPD) technique as previously described [31]. Seminiferous tubules were fixed in 2% OsO_4_ in bidistilled water at room temperature for 45 min and rinsed in 6.5% sucrose in distilled water for 30 min. During dehydration in ethanol the material was treated with 4% p-phenylenediamine (PPD) (Merck) in 70% ethanol for 2 hours at room temperature. Then, testes were rinsed in 70% ethanol, dehydrated and embedded in Epon 812. Thin sections (0.2–0.5 μm) were obtained in a Reichert-Jung ultramicrotome, transferred to copper/rhodium grids, and observed without posterior contrasting. All observations were made in a Jeol 1010 transmission electron microscope operated at 80 kV.

## Abbreviations

CENP-A: centromeric protein A
CLiP: chromatid linking protein
ICIS: inner centromere kin/stimulator
INCENP: inner centromeric protein
kMT: kinetochore microtubule
LE: lateral element of the synaptonemal complex
MCAK: mitotic centromere-associated kinesin
MT: microtubule
Os-PPD: osmium tetroxide/p-phenylenediamine technique
RAD: radiance sensitive protein
SGO: shugoshin
SYCP3: synaptonemal complex protein 3
